# *Santolina pinnata* Viv. Exerts Promising Antitumor Activity against Breast Cancer Cells and Anti-Inflammatory Effects in LPS-Stimulated RAW 264.7 Cells

**DOI:** 10.3390/ijms232112885

**Published:** 2022-10-25

**Authors:** Matteo Brindisi, Luca Frattaruolo, Vincenzo Sicari, Monica Rosa Loizzo, Gianni Bedini, Vittoria Rago, Rosa Tundis, Anna Rita Cappello

**Affiliations:** 1 Cell Adhesion Unit, San Raffaele Vita-Salute University, 20132 Milan, Italy; 2 Department of Pharmacy, Health and Nutritional Sciences, University of Calabria, 87036 Rende, Italy; 3 Department of Agraria, “Mediterranea” University of Reggio Calabria, 89122 Reggio Calabria, Italy; 4 Department of Biology, University of Pisa, 56122 Pisa, Italy

**Keywords:** *Santolina pinnata*, phytochemicals, breast cancer, anticancer activity, anti-inflammatory

## Abstract

Cancer is one of the largest causes of mortality in the world, and due to its incidence, the discovery of novel anticancer drugs is of great importance. Many successful anticancer drugs used in clinical practices are derived from natural products. The genus *Santolina* is a group of species distributed in the Mediterranean area and used in traditional medicine for their biological properties. The aim of this work was to investigate, for the first time, the multi-target biological potential of Italian *Santolina pinnata* in relation to their chemical profile, by which an interesting natural source of valuable phytochemicals endowed with anticancer and anti-inflammatory features could be assessed. *n*-Hexane (EHSP) and methanol (EMSP) extracts were investigated by gas chromatography (GC) and gas chromatography-mass spectrometry (GC-MS) and ultra-high-performance liquid chromatography (UHPLC), respectively. Anti-proliferative activity was analyzed on MCF-7 and MDA-MB-231 breast cancer cells, as well as on non-tumorigenic MCF-10A cells, by the 3-(4,5-dimethylthiazol-2-yl)-2,5-diphenyl-2H-tetrazolium bromide (MTT) assay. Apoptotic death was assessed by comet assay. Cell motility and invasive features were examined in highly invasive MDA-MB-231 by wound-healing scratches, while, in both breast cancer cell lines, by gel-zymography experiments. The anti-inflammatory potential was analyzed by nitric oxide (NO) production and the nuclear factor kappa-light-chain-enhancer of activated B cells (NF-κB) staining experiments in *bacterial* lipopolysaccharides (LPS) which stimulated RAW 264.7 cells. EHSP and EMSP extracts exhibited anticancer activity against breast cancer cells, promoting apoptotic death, as well as decreasing cell migration and invasive behaviours. The highest activity (IC_50_ of 15.91 μg/mL) was detected against MDA-MB-231 cells, a highly invasive breast cancer cell line. Both extracts were also able to promote anti-inflammatory effects (IC_50_ values ranging from 27.5 to 61.14 μg/mL), as well as to reduce NO levels by inducing inhibitory effects on NF-κB nuclear translocation in LPS-stimulated RAW 264.7 cells. The different biological behaviours found between the extracts could be related to their different chemical compositions. Herein, the multi-target biological potential of *S. pinnata* in inducing antitumor and anti-inflammatory effects was comprehensively demonstrated. These findings will provide important stepping-stones for further investigations and may lead to the development of highly effective *S. pinnata* extract-based treatments for breast cancer and inflammatory processes.

## 1. Introduction

Breast cancer is the most common type of cancer and one of the major causes of cancer death in women. In 2020, breast cancer recorded high global incidence and mortality rates [1]. Despite the progress achieved in technology that has enabled the early detection of breast cancer, about 30% of patients with early stage cancer eventually relapse with metastases [2,3]. Metastatic breast cancer is considered to be generally incurable, with a 5-year survival rate of only 26% despite now available treatment opportunities [4]. Based on the receptor status, four major molecular subtypes of breast cancer have been identified, including Human Epidermal Growth Factor Receptor 2 (HER2)-enriched, triple-negative, and luminal A/B subtypes [5]. Each subtype exhibits distinct biological features, with variability in both prognosis and treatment response [6,7,8]. A combination of surgery, endocrine therapy, radiotherapy, chemotherapy, or HER2-targeted therapy is often employed in the treatment plan depending on the subtype and stage of breast cancer, as well as the tolerance of patients [9]. Unfortunately, there have been reports of resistance to endocrine therapy, HER2-targeted therapy, and chemotherapy. In recent decades, cancer research has focused on identifying innovative and effective therapeutic strategies aimed at overcoming resistance to conventional therapies phenomena and hit triple-negative breast cancer (TNBC). Although recent studies allow new therapeutic regimes to be approved [10,11,12], the search for new natural approaches characterized by high efficacy and minimal toxicity is interesting in the breast cancer field.

Nature has always served as a rich source of chemical compounds with promising anti-cancer effects [13]. More than three thousand species containing active phytochemicals have been reported to possess anti-cancer properties, and about thirty plant-derived compounds, mainly including several phenolic compounds, flavonoids, alkaloids, and terpenes as main classes of molecules, have been isolated and tested in clinical trials [14]. Most of these compounds demonstrated an ability to inhibit cell proliferation and induce apoptosis and/or growth arrest by targeting different cellular signaling pathways.

In this regard, in our previous study, we investigated the anti-proliferative activity of *Santolina corsica*. Extracts showed a promising inhibition in the motility, migration, and invasion rate of a highly invasive breast cancer cell line, MDA-MB-231 [15].

Following these promising results, in the present work, we investigated for the first time the anti-proliferative and anti-inflammatory activity of another *Santolina* species, *Santolina pinnata*. *Santolina* (Asteraceae) is a genus represented by 26 species (POWO, 2022) commonly in the Mediterranean area but taxonomically complex, whose classification is periodically revised [16].

With the increasing interest in the search for bioactive compounds from the Santolina species, in recent years, phytochemical studies have revealed the presence of terpenes, including monoterpenes, eudesmane-type sesquiterpenes, germacrene-type sesquiterpenes, dammarane-type triterpenes, coumarins, and flavonoids as the main classes of constituents. The *Santolina* species have shown to possess promising biological activities, including antiviral, anti-inflammatory, antibacterial, antifungal, hepatoprotective, and cytotoxic effects [15,16].

*Santolina pinnata* Viv. is a subshrub with a woody stem, herbaceous naked, and glabrous flowering shoots. The leaves reach up to the lower half of the flowering shoots and are arranged in two opposite or four crossing rows, linear (4.5 cm) and pinnate, with a pinnae of up to 6 mm. A single, spherical flower head (capitulum, 6–10 mm in diameter) is borne on top of each of the flowering shoots. Corollas are white to off-white, bell-shaped, with five acute triangular lobes inserted on a convex receptacle. The fruits (cypselas) are oblong, three-angled, and without pappus. It is endemic to the Apuan Alps (Northern Tuscany, Central Italy), where it grows on limestone cliffs, screes, and pavements from 500 to 1500 m asl. The populations are numerous, and flowers are abundant in July. The species’ conservation status was assessed as LR according to the 1994 criteria [17]. It is included in the *S. chamaecyparissus* species complex [17] and interpreted as a schizo-endemic unit of a group of related diploid (2*n* = 18) peninsular entities (*S. ligustica* Arrigoni, *S. etrusca* (Lacaita) Marchi, S. neapolitana Jord. & Fourr.) [18]. It is locally known as “crespolina apuana”, “pan di capra”, and “camomilla selvatica”.

Herein, we investigate the anti-cancer effects and possible underlying mechanisms of two *S. pinnata* extracts on two breast cancer cell lines, MCF-7, *estrogen* receptor-α positive (ERα+), and MDA-MB-231, TNBC. Moreover, since the inflammatory process is closely related to the onset and development of many cancers, we also evaluated, for the first time, the anti-inflammatory potential of S. pinnata extracts in order to consider them as potential adjuvants in therapeutic applications.

## 2. Results and Discussion

### 2.1. Identification of Potential Active Compounds

UHPLC analysis of the methanol extract of *S. pinnata* led to the identification of some flavonoids and phenolic compounds. The data are reported in Table 1. Chlorogenic acid and rosmarinic acid are the most abundant identified phenolic compounds (928.1 and 591 mg/100 g, respectively), while the dominant flavonoids are rutin (434.3 mg/100 g), genistein (271.6 mg/100 g), and myricetin (211.6 mg/100 g). Apigenin, kaempferol, and luteolin are found only in trace quantities.

Previously, only one work analysed the chemical profile of *S. pinnata*. In this study, Flamini et al. [19] described the presence of the flavonoids apigenin, luteolin, chrysoeriol, luteolin-7-glucoside, nepetin, kaempferol, and pectolinarigenin in the aerial parts of *S. pinnata* from Italy.

The analysis of the *n*-hexane extract led to the identification of 38 main constituents (Table 2). Monoterpene hydrocarbons represent 12.4% of the total extract, followed by sesquiterpene hydrocarbons (11.2%).

Other compounds represent 48.4% of the extract and include alkanes, aldehydes, fatty acids, and their derivatives. Among monotepene hydrocarbons, the most abundant compounds are myrcene (3.2%), γ-terpinene (2.3%), sabinene (1.7%), and β-pinene (1.6%). Among the oxygenated monotepenes the dominant volatiles, are 1,8-cineole (2.7%), terpinene-4-ol (2.3, and canphor (1.2%).

(-)-Bornyl acetate, γ-cadinene, and δ-cadinene (4.3, 3.2%, and 1.8%, respectively) are the most abundant sesquiterpene hydrocarbons. Two diterpenes, neophytadiene and phytol, and one oxygenated sesquiterpene, spathulenol were identified.

### 2.2. S. pinnata Showed Interesting Anti-Proliferative Effect against Breast Cancer

Neoplastic pathologies are a great issue of public health and still constitute a major cause of death worldwide. Surgery, radiation, and chemotherapy act as useful treatments in this field, and thus, this research is aimed at discovering new chemotherapeutic agents to counter this severe question.

Plants are a great source of compounds endowed with various biological activities [15,20,21]. Having an extraordinary diversity in nature, plants are recognized as rich sources of bioactive molecules. Over the past decades, many efforts have been made to isolate new compounds from natural sources like plants, marine organisms, and microorganisms to develop anti-cancer agents. Since 1981, about 25% of new anti-cancer drugs have been derived from natural sources. Therefore, bioactive compounds, isolated from natural sources, can be utilized to fulfill the continuous request for anticancer agents.

The interesting therapeutic potential of the genus *Santolina* (Asteraceae) fits into this context, as evidenced by several recent reports [15,16,22,23]. On this basis, herein, we evaluated, for the first time, the antitumor potential of the methanol (EMSP) and n-hexane (EHSP) extracts of *S. pinnata* on MCF-7 and MDA-MB-231 breast cancer cells. We used cell lines characterized by important differences in the three main receptors conventionally used for breast cancer subtyping: estrogen receptor (ER), progesterone receptor (PR), and human epithelial receptor (HER 2). In particular, MCF-7 cells typified by the presence of ERα (ERα +) and MDA-MB-231 were identified as TNBC.

**Figure 1:**
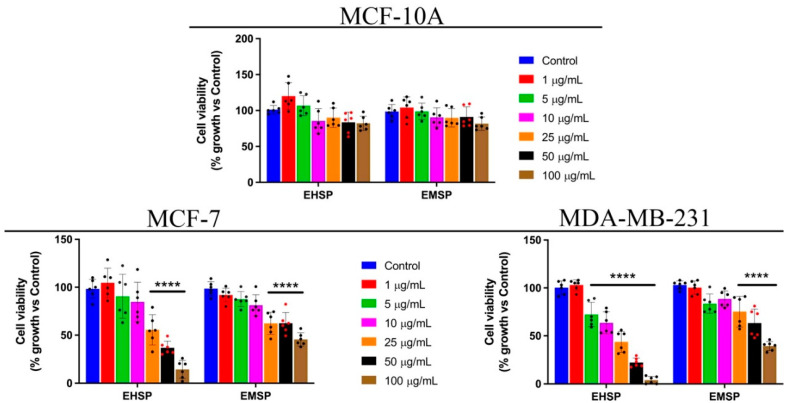
MCF-10A, MCF-7 and MDA-MB-231 viability after EHSP or EMSP treatments (from 100 to 1 μg/mL), for 72 h. MTT assay was used to assess cell viability. Values represent means ± SD from three independent experiments (*n* = 6). Statistical analysis was performed using one-way ANOVA analysis. **** *p* < 0.0001.

The effects of the two extracts were also assessed on MCF-10A, a normal breast epithelial cell line. A wide range of concentrations (100, 50, 25, 10, 5, and 1 µg/mL) of EHSP and EMSP was used to evaluate cell viability after 72 h of treatment by the MTT assay. As shown in Figure 1, we found that both extracts elicited a dose-dependent inhibition of cell viability of the two tested breast cancer cell lines. Surprisingly, the best results were obtained on the aggressive TNBC cell line, especially after EHSP treatment.

In addition, both extracts did not manifest toxic effects on the non-tumorigenic cell line, underlining a selective activity towards tumor cells. In detail, the EHSP extract was more active on both breast cancer cell lines with IC_50_ values of 31.61 μg/mL and 15.91 μg/mL in MCF-7 and MDA-MB-231, respectively (Table 3). On the other hand, the EMSP extract displayed almost equal activity against both breast cancer lines analyzed, with IC_50_ values of 65.22 and 72.54 μg/mL in the MCF-7 and MDA-MB-231, respectively (Table 3).

We speculate that the different behaviors displayed by the two extracts could be related to their different chemical compositions; indeed, from Table 1 and Table 2, it can be seen that the EHSP extract includes multiple constituents, in particular monoterpenes with known anticancer activity, such as myrcene, γ-terpinene, and 1,8-cineole. These phytochemicals, generally recognized as safe (GRAS) by the Food and Drug Administration (FDA, USA), have been demonstrated to be promising anti-tumor agents in both in vitro and in vivo studies through the promoting of apoptosis, autophagy, and cell cycle arrests with mechanisms of action that include the modulation of oxidative stress, the modulation of the endoplasmic reticulum stress inhibition of proteins involved in cell survival proliferation, and the depolarization of mitochondrial membrane potential [24]. Moreover, their ability to increase anti-cancer activity in combination with known anti-neoplastic drugs was reported [24].

Furthermore, the results concerning the composition and IC_50_ values of the EHSP extracts are in line with those we previously obtained with the *n-hexane* extract of *S. corsica* [15] tested against the same breast cancer cells. Even in that case, the methanol extract was less rich in components with antitumor activity and, therefore, less active. Taken together, our findings highlight the anticancer potential of the genus *Santolina*.

### 2.3. EHSP and EMSP Induce Apoptotic Death in Breast Cancer Cells

**Figure 2:**
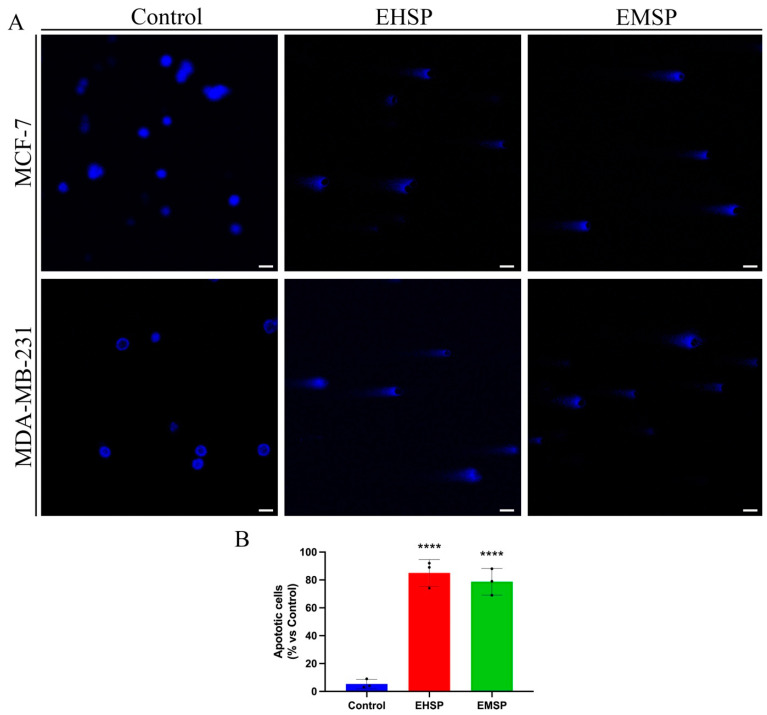
(**A**) Comet assay of MCF-7 and MDA-MB-231 cells treated with EHSP or EMSP at their IC_50_ values, or with the vehicle alone (Control) for 3 days. 4′,6-Diamidino-2-phenylindole dihydrochloride,2-(4-Amidinophenyl)-6-indolecarbamidine dihydrochloride (DAPI) was used to stain nuclei. Pictures were obtained at 20×. Scale bar 25 µm. (**B**) Apoptotic cells quantification. Values represent means ± SD from three independent experiments (*n* = 3). Statistical analysis was performed using one-way ANOVA analysis. **** *p* < 0.0001.

Since our findings have shown promising anti-proliferative activity of the EHSP and EMSP extracts on the MDA-MB-231 cell line, typified by a high aggressiveness and absence of specific targeted pharmacological therapies that correlates them to a lower relapse-free survival (RFS) [25], we then asked if the anti-proliferative activity elicited by both extracts (especially EHSP) was related to apoptotic death. For this purpose, MCF-7 and MDA-MB-231 cells were treated, for 72 h, with both extracts, at their IC_50_ values or with the vehicle alone (Control), and subsequently analyzed by the comet assay (Figure 2) in order to verify any DNA fragmentation at the late event of the apoptotic process.

### 2.4. EHSP and EMSP Reduce Motility and Infiltrating Power of Breast Cancer Cells

**Figure 3:**
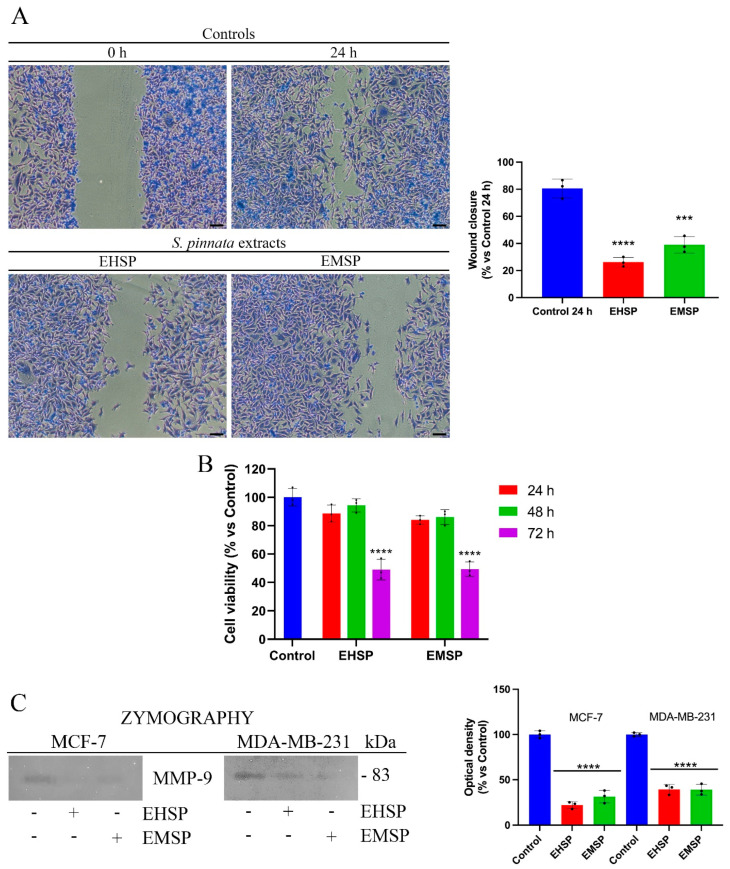
(**A**) Wound healing of MDA-MB-231 cells exposed to EHSP or EMSP, at IC_50_ values, for 24 h. Pictures were taken at 10×. Scale bars 50 μm. Histogram represents the wound closure quantification. (**B**) Viability of MDA-MB-231 cells exposed to EHSP or EMSP, at IC_50_ values, for 24, 48, and 72 h. (**C**) Gel-zymography of MCF-7 and MDA-MB-231 exposed to EHSP or EMSP at IC_50_ values for 24 h. MCF-7 cells were pre-treated with 80 ng/mL PMA for 24 h. Histogram represents gel-zymography quantification in MCF-7 and in MDA-MB-231. Values represent means ± SD from three independent experiments (*n* = 3). Statistical analysis was performed using one-way ANOVA analysis. *** *p* < 0.001; **** *p* < 0.0001.

MDA-MB-231 cells, unlike MCF-7, are characterized by a high aggressiveness due to marked motility and invasiveness [26]. In fact, their feature is to move quickly and release large amounts of metalloproteinases, in particular MMP-9, which are able to digest ECM and invade the ECM with the consequent invasion of the surrounding tissues. Based on this, we used MDA-MB-231 cells as a model to evaluate any effects of EHSP and EMSP extracts on cell motility. To this end, we firstly confirmed the high motility of MDA-MB-231 cells by the wound healing assay (Figure 3A), with untreated cells managing to close the wound in about 24 h (Control). At the same time, after treatments with EHSP and EMSP, the cells clearly highlighted a decreased migratory power (Figure 3A). The concentration of the extracts used were the IC_50_ values calculated after 72 h of treatment. As highlighted by Figure 3B, these concentrations did not affect cell viability after 24 h, which was the wound healing assay endpoint. This result was also supported by a reduction in the *Matrix Metalloproteinase-9* (MMP-9) secretion and activity; as displayed in Figure 3C, through gel-zymography experiments, we proved that decreased levels of MMP-9 secretion occur in MDA-MB-231 cells, after EHSP or EMSP exposure at their IC_50_ values. Furthermore, we also performed Gel-zymography experiments on MCF-7 cells.

However, due to the reduced or completely absent motility (for this reason, the wound healing assay was not performed) of these cells as well as their low infiltrating power as they were able to release low levels of MMP-9, we pre-treated them with 80 ng/mL phorbol 12-myristate 13-acetate (PMA), a pro-tumor factor involving metalloproteinases modulation [27], in order to obtain detectable MMP-9 values. Then, MCF-7 cells were exposed to EHSP or EMSP at the respective IC_50_ values. Our results highlighted that EHSP or EMSP treatment induces significant reductions in the MMP-9 levels (Figure 3C), confirming the potential of the extract to reduce motility and invasiveness in both the breast cancer lines analyzed.

### 2.5. Anti-Inflammatory Activity of S. pinnata

The strong link between the inflammatory process and tumor onset/development is widely reported in the literature [28]. In fact, several pieces of reported evidence confirm that an inflammatory process, tending to persist, can lead to the subversion of the tissue, driving neoplastic transformation [28]. Similarly, in recent years, different evidence has described how the tumor exploits the inflammatory process to develop [28,29]. It is, therefore, interesting to identify new compounds endowed with both antitumor and anti-inflammatory activities.

**Figure 4:**
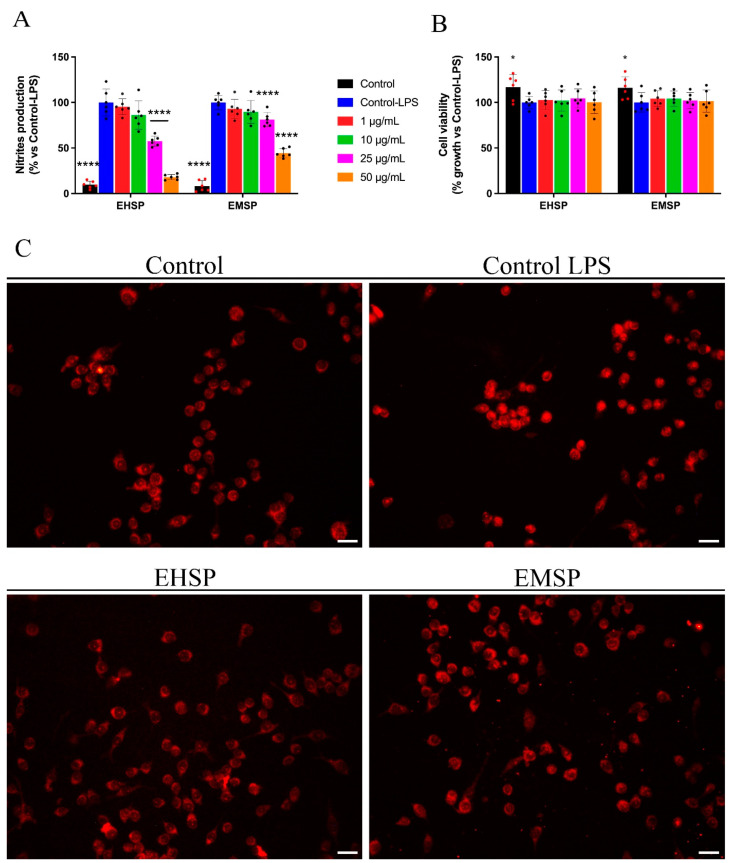
Nitrites production (**A**) and cell viability (**B**) were assessed by Griess assay and MTT assay, respectively, after treatment with LPS-stimulated RAW 264.7 cells with EHSP or EMSP at different concentrations (from 50 µg/mL to 1 µg/mL), for 24 h (*n* = 6). (**C**) Immuno-fluorescent localization of NF-κB in RAW 264.7 cells treated for 1 h with DMSO or 1 µg/mL LPS + DMSO (Control and Control LPS, respectively), 1 µg/mL LPS EHSP or EMSP (at their IC_50_ values, respectively) (*n* = 3). Scale bar: 50 µm. Values represented as mean ± S.D. of three independent experiments. Statistical analysis was performed using one-way ANOVA analysis. * *p* value < 0.05; **** *p* < 0.0001.

Given the previous reports that highlight the promising anti-inflammatory activity of the genus *Santolina*, particularly of *S. corsica* [15], herein, for the first time, we assessed the anti-inflammatory effect of both methanolic and n-hexane extracts of *S. pinnata*. Therefore, we evaluated by the Griess assay NO production, a key mediator of the inflammatory process, in LPS-stimulated RAW 264.7 cells, a validated cell model for the in vitro study of the anti-inflammatory potential of bioactive compounds. For this purpose, cells were treated with increasing amounts (50, 25, 10, and 1 μg/mL) of EHSP and EMSP extracts or with the vehicle alone (Control) for 24 h. Both extracts dose-dependently inhibited NO production (Figure 4), and the best activity was scored by the EHSP extract with an IC_50_ value of 27.5 μg/mL; conversely, the EMSP extract was able to inhibit NO production at the highest concentration with an IC_50_ value of 61.14 μg/mL. The tested concentrations of both extracts did not show any effect on the cell viability of RAW 264.7 cells (Figure 4).

As already reported for the *S. corsica* extracts [15], the effects highlighted for the *S. pinnata* extracts suggest that their different activity was due to the different chemical compositions of the extracts. Indeed, as the n-hexane extract of *S. corsica*, the homologue of *S. pinnata* includes many compounds endowed with known anti-inflammatory activities that justify its remarkable activity. At the same time, contrary to the methanol extract of *S. corsica*, devoid of anti-inflammatory activity, the slightest activity displayed from the homologue of *S. pinnata* can be due to myricetin, a common plant flavonoid endowed with known anti-inflammatory activity and, for the first time, highlighted in an extract of *S. pinnata*. NO production was mediated by the enzymatic activity of the inducible nitroxide synthase (iNOS) enzyme, whose expression was regulated by the nuclear factor kappa-light-chain-enhancer of activated B cells (NF-κB), which is the main transcription factor involved in the inflammatory response.

Based on Griess’s results, we evaluated the ability of EHSP and EMSP to interfere with the signaling mediated by NF-κB and, in particular, to evaluate whether the two extracts were able to modulate the activity of this transcription factor. In detail, LPS-stimulated RAW 264.7 cells, after treatment with EHSP and EMSP at their respective IC_50_ values, were subjected to the immunofluorescence assay in order to evaluate any effects on the cytosol-nucleus translocation of NF-κB.

The results obtained, as shown in Figure 4, highlight that in LPS-stimulated RAW 264.7 cells, nuclear localization of NF-κB took place, compared to the non-stimulated cells in which a clear cytosolic localization was evident, confirming that the inflammatory stimulus favors the nuclear translocation of NF-κB and induces its consequent transcriptional activity. At the same time, EHSP and EMSP treatments of LPS-stimulated RAW 264.7 cells showed a clear reduction of NF-κB nuclear translocation with respect to that assessed in untreated stimulated cells. This result confirms previous evidence concerning the NO production decrease, suggesting that the effect on NO levels may be related to their involvement in the NF-κB pathway.

## 3. Materials and Methods

### 3.1. Chemicals and Reagents

Solvents used in this study were obtained from VWR International s.r.l. (Milan, Italy). Apigenin, caffeic acid, chlorogenic acid, (-)-epicatechin, ferulic acid, gallic acid, genistein, kaempferol, luteolin, myricetin, protocatechuic acid, quercetin-3-*O*-glucoside, rosmarinic acid, rutin, and vanillic acid were purchased from Sigma Aldrich (Milan, Italy). All the reagents needed for cell culture were purchased from Sigma-Aldrich (Milan, Italy), together with dimethyl sulfoxide (DMSO, cat.n. D8418), 3-(4,5-dimethyl-2-thiazolyl)-2,5-diphenyl-2H-tetrazolium bromide (MTT, cat.n. M2128), LPS (cat.n. L2630), DAPI (cat.n. D9542), Coomassie brilliant blue (cat.n. B0770), and the Griess reagent (cat.n. G4410).

### 3.2. Plant Materials and Extraction Procedure

The aerial parts of *S. pinnata* were collected in July 2018 from a large population on the Antona mountain, Piano della Fioba (MS), Italy, at an altitude of 900 m on limestone cliffs (dolomite) exposed to SSW, by Gianni Bedini, University of Pisa, Italy.

*S. pinnata* aerial parts were exhaustively extracted with *n*-hexane and methanol (140 and 135 g, respectively) by maceration (3 × 72 h) to give 11.3 and 1.4 g of the respective dried residue with extraction yields of 8.1 and 1.0%, respectively.

### 3.3. Chemical Analyses

The methanol extract of *S. pinnata* was analysed by q ultra-high-performance liquid chromatography (UHPLC) system consisting of a UHPLC PLATINblue (Knauer, Berlin, Germany) equipped with a binary pump system and using a Knauer Blue Orchid column C18A (1.8 μm, 100 mm × 2 mm) coupled with a PDA-1 (Photo Diode Array Detector) PLATINblue (Knauer, Berlin, Germany).

Compounds were quantified by the direct injection of the sample, dissolved in methanol, and filtered with 0.22 μm PTFE syringe filters, with a diameter of 13 mm (Thermo Fischer Scientific, Waltham, MA, USA). The analysis required a flow rate of 0.4 mL/min, a column temperature of 30 °C, and an injection volume of 5.0 μL. Water/formic acid 0.2% (A) and acetonitrile (B) were mobile phases, and the gradient program was the following 0–3 min, 95% A and 5% B; 3–15 min, 95–60% A and 5–40% B; 15–15.5 min, 60–0% A and 40–100% B. Based on the literature data, apigenin, caffeic acid, chlorogenic acid, (-)-epicatechin, ferulic acid, gallic acid, genistein, kaempferol, luteolin, myricetin, protocatechuic acid, quercetin-3-*O*-glucoside, rosmarinic acid, rutin, and vanillic acid were chosen as markers and analyzed. Identification and quantification were carried out based on recorded retention times in comparison with authentic standards at 280, 254, 330, and 305 nm. A calibration straight for each standard was obtained by analysing the standard solution diluted at different concentrations, and the results were elaborated with the Clarity 6.2 software.

The *n*-hexane extract of *S. pinnata* was analysed by gas chromatography associated with mass spectrometry (GC-MS) by using a Hewlett-Packard gas chromatograph (Agilent, Milan, Italy) associated with a Hewlett-Packard mass spectrometer (Agilent, Milan, Italy) and equipped with an HP-5 capillary column (30 m × 0.25 mm, 0.25 μm) and by using a Shimadzu GC17A gas chromatograph equipped with an ionization flame detector (GC-FID) and an HP-5 capillary column (30 m × 0.25 mm, 0.25 μm) (Shimadzu, Milan, Italy), as previously described [15].

### 3.4. Cell Cultures

MCF-7, MDA-MB-231, MCF-10A, and RAW 264.7 cells were cultured as previously described [15].

### 3.5. Cell Viability

Cell viability of MCF-7, MDA-MB-231, MCF-10A, and RAW 264.7 cells was assessed by the MTT assay as previously described [30].

### 3.6. Comet Assay

The comet assay of MCF-7 and MDA-MB-231cell lines was assessed as previously described [31].

### 3.7. Scratch and Gel-Zymography Experiments

The scratch assay on MDA-MB-231 was assessed as previously described [30]. Gel-zymography experiments were assessed on the conditioned media from MCF-7 or MDA-MB-231. Briefly, MCF-7 and MDA-MB-231 cells were grown in 24-well plates, then washed twice with PBS and treated (in FBS-free media) for 24 h with EHSP or EMSP at their IC_50_ values, respectively. At the end of treatment, the conditioned media was collected and centrifuged to eliminate dead cells. Samples were added with a 5× non-reducing sample buffer (4% SDS, 20% glycerol, 0.01% bromophenol blue, and 125 mM Tris-HCL, pH 6.8), loaded into the gelatin-acrylamide gel and exposed to a 150 V electrophoresis run.

Then, the gel was washed twice, for 30 min each, in a washing buffer (2.5% Triton X-100, 50 mM Tris-HCL, pH 7.5, 5 mM CaCl_2_, 1 μM ZnCl_2_). At the end of the washing procedures, the gel was rinsed with the incubation buffer (1% Triton X-100, 50 mM Tris-HCL, pH 7.5, 5 mM CaCl_2_, 1 μM ZnCl_2_) and left at 37 °C, for 24 h. After 24 h of incubation, the gel was stained in a Coomassie blue solution and then de-stained until the bands could be clearly seen. The gel was then scanned, and band densities were quantified using ImageJ software.

### 3.8. Griess and NF-κB Immunofluorescence Experiments

The Griess assay on LPS-stimulated RAW 264.7 cells treated with EHSP or EMSP was assessed as previously described [15]. The nuclear translocation of NF-κB was assessed as previously described [32].

### 3.9. Statistical Analysis

The GraphPad Prism version 9.2.0 (GraphPad Software, San Diego, CA, USA) was used to calculate the concentration giving 50% inhibition (IC_50_). In the biological tests, differences within and between the groups were evaluated by the one-way analysis of variance test (ANOVA) followed by a multi-comparison Dunnett’s test. In the chemical analysis, Tukey’s test was used to determine any significant difference in chemical parameters among the investigated samples.

## 4. Conclusions

Taken together, our findings corroborate the anti-proliferative activity of the genus *Santolina* in MCF-7 and MDA-MB-231 cell lines, underlining their selectivity towards tumor cells. On the other hand, for the first time, they highlighted the ability of both *S. pinnata* extracts to trigger apoptotic death in breast cancer cells, as well as to reduce motility and the infiltrating power of the aggressive TNBC cell line, emphasizing their potential in decreasing the metastatic ability of these tumor cells, which still remain the major responsibility for therapy failing.

Furthermore, herein, for the first time, we established that *n*-hexane and methanol *S. pinnata* extracts exert anti-inflammatory effects by interfering with the inflammatory cascade mediated by NF-kB in LPS-activated macrophages. On this basis, they might counteract chronic inflammation so as to decrease the incidence of many inflammatory-derived diseases. However, later insights will be needed in order to better understand the exact molecular targets responsible for the anti-inflammatory action.

## Figures and Tables

**Table 1:** The main identified compounds in the methanol extract of *S. pinnata* (EMSP).

Compound	mg/100 g
***Phenolics***	
Chlorogenic acid	928.1 ± 3.3
Caffeic acid	42.3 ± 1.3
Ferulic acid	129.6 ± 2.1
Gallic acid	42.7 ± 0.8
Rosmarinic acid	591.0 ± 4.2
Vanillic acid	6.5 ± 0.4
***Flavonoids***	
Apigenin	tr
(-)-Epicatechin	171.8 ± 3.2
Genistein	271.6 ± 4.2
Kaempferol	tr
Luteolin	tr
Myricetin	211.6 ± 2.5
Protocatechuic acid	81.9 ± 1.0
Quercetin-3-*O*-glucoside	150.9 ± 1.3
Rutin	434.3 ± 3.3

Data are reported as mean ± standard deviation (*n* = 3). tr: traces.

**Table 2:** The main identified constituents (%) of *S. pinnata n*-hexane extract (EHSP).

Compound	RI ^a^	Content (%)	M ^b^
***Monoterpene hydrocarbons***			
α-Pinene	938	0.5 ± 0.02	a,b,c
Camphene	953	0.7 ± 0.03	a,b,c
Sabinene	973	1.7 ± 0.08	a,b,c
β-Pinene	980	1.6 ± 0.2	a,b,c
Myrcene	993	3.2 ± 0.1	a,b,c
α-Phellandrene	1005	0.2 ± 0.01	a,b
α-Terpinene	1012	0.2 ± 0.01	a,b,c
*p*-Cymene	1025	0.3 ± 0.07	a,b
Limonene	1030	0.5 ± 0.06	a,b,c
γ-Terpinene	1057	2.3 ± 0.7	a,b,c
Terpinolene	1086	1.2 ± 0.01	a,b,c
***Oxygenated monoterpenes***			
1,8-Cineole	1034	2.7 ± 0.4	a,b,c
Linalool	1098	0.3 ± 0.02	a,b,c
Camphor	1145	1.2 ± 0.76	a,b
Borneol	1167	1.1 ± 0.08	a,b
Terpinen-4-ol	1176	2.3 ± 0.06	a,b
***Sesquiterpene hydrocarbons***			
(-)-Bornyl acetate	1286	4.3 ± 0.12	a,b
α-Cubebene	1352	0.6 ± 0.03	a,b
α-Copaene	1377	0.2 ± 0.02	a,b
*trans*-Caryophyllene	1415	0.7 ± 0.04	a,b
*allo*-Aromadendrene	1462	0.4 ± 0.01	a,b
γ-Cadinene	1515	3.2 ± 0.05	a,b
δ-Cadinene	1526	1.8 ± 0.01	a,b
***Oxygenated sesquiterpenes***			
Spathulenol	1578	1.2 ± 0.06	a,b
***Diterpenes***			
Neophytadiene	1830	1.7 ± 0.06	a,b
Phytol	1950	1.1 ± 0.02	a,b
***Other compounds***			
Octadecane	1800	4.5 ± 0.9	a,b,c
Hexadecanal	1811	0.7 ± 0.02	a,b
Nonadecane	1900	1.6 ± 0.01	a,b,c
Methyl palmitate	1934	0.6 ± 0.02	a,b
Palmitic acid	1969	2.4 ± 1.1	a,b
Eicosane	2000	3.9 ± 0.01	a,b,c
Octadecanal	2021	5.4 ± 0.01	a,b
Linoleic acid	2156	1.5 ± 0.03	a,b
Pentacosane	2500	4.9 ± 1.21	a,b,c
Heptacosane	2700	6.8 ± 1.1	a,b,c
Octacosane	2800	3.7 ± 1.2	a,b,c
Nonacosane	2900	12.4 ± 2.3	a,b,c
***Total identified***		83.6	

Data are reported as the mean ± standard deviation (*n* = 3). ^a^ RI: Retention indices on the HP5 column. ^b^ M, identification method: a. Comparison of retention times, b. Comparison of mass spectra with MS libraries, c. Comparison with authentic compounds; tr: trace (<0.1%).

**Table 3:** Cytotoxic activity of *n*-hexane (EHSP) and methanol (EMSP) extracts.

Cell Line	IC_50_ (μg/mL)	
	EHSP	EMSP
MCF-10A	>100 / ^(a)^	>100 / ^(a)^
MCF-7	31.61 23.63 to 42.34 ^(a)^	65.22 47.48 to 90.88 ^(a)^
MDA-MB-231	15.91 12.96 to 19.86 ^(a)^	72.54 57.70 to 92.05 ^(a)^

^a^ 95% confidence intervals.

## Data Availability

Not applicable.
